# Response to “Multi-omics analysis of druggable genes to facilitate Alzheimer's disease therapy: A multi-cohort machine learning study”

**DOI:** 10.1016/j.tjpad.2025.100250

**Published:** 2025-06-27

**Authors:** Jichang Hu

**Affiliations:** Department of Pathophysiology School of Basic Medicine Key Laboratory of Education Ministry/Hubei Province of China for Neurological Disorders Tongji Medical College, Huazhong University of Science and Technology, Wuhan, China

Dear Carine,

Thank you for your email regarding the understanding of the issues in Fig. 8A and B of our manuscript.

**Fig. 8:**
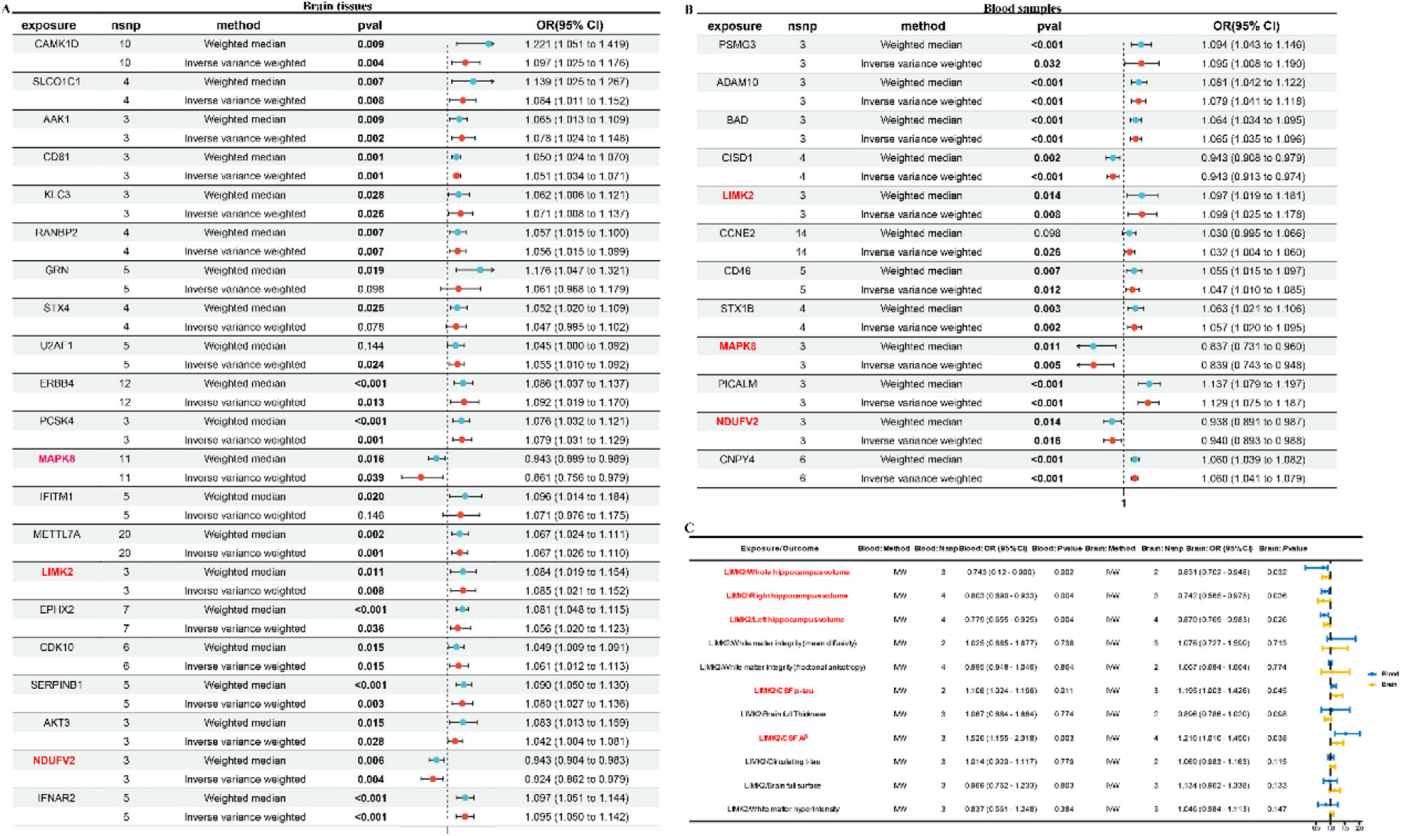
Identification of hub genes in AD by MR. (A) Forest plot for MR results between brain eQTL and AD. (B) Forest plot for the MR result between blood eQTL and AD. (C) MR results of AD markers and AD outcome in IVW method. (Image source: Hu et al. [1], Multi-omics analysis of druggable genes to facilitate Alzheimer's disease therapy: A multi-cohort machine learning study).

As Yin et al. mentioned, an OR greater than 1 implies that an increase in the genetically predicted exposure level is causally associated with an elevated risk of binary outcomes, indicating that the exposure serves as a risk factor for the outcome. Conversely, an OR less than 1 indicates a causal relationship between an increase in the genetically predicted exposure level. We partially agree with their point of view. Actually,

in Mendelian randomization (MR) analyses, an OR value greater than 1 with a p-value less than 0.05 generally indicates that the gene is associated with an increased risk of the disease. Whether to consider gene expression levels depends on the specific objectives and context of the study. If the research aims to delve into the biological mechanisms linking the gene to the disease, then taking gene expression into account may be meaningful. However, if the primary focus is to assess causal relationships, then gene expression levels may not need to be considered.

In our current study, we found LIMK2 OR 1.084 (1.019–1.154) with a p-value < 0.05, MAPK8 OR 0.943 (0.899–0.989) with a p-value < 0.05, and NDUFV2 OR 0.943 (0.904–0.983) with a p-value < 0.05. Therefore, we hastily drew the conclusion - LIMK2, MAPK8, and NDUFV2 might contribute to an enhanced risk of AD by MR in brain tissues and blood samples [1]. Analysis of gene expression profiles will enable more precise elucidation of their biological relevance. It is the limitation of our manuscript.

Best regards,

Jichang

## CRediT authorship contribution statement

**Jichang Hu:** Writing – original draft, Writing – review & editing.

## Declaration of competing interest

The authors declare that they have no known competing financial interests or personal relationships that could have appeared to influence the work reported in this paper.

## References

[1] Hu J., Luo Y., Wang X. (2025). Multi-omics analysis of druggable genes to facilitate Alzheimer's disease therapy: a multi-cohort machine learning study. J Prev Alzheim Dis.

